# A study of Inter-Technology Information Management (ITIM) system for industry-education integration

**DOI:** 10.1016/j.heliyon.2023.e19928

**Published:** 2023-09-07

**Authors:** Zhenhua He, Lifeng Chen, Lianqin Zhu

**Affiliations:** a School of Logistics and Transportation and Tourism, Jiangsu Vocational College of Finance and Economics, Huaian, 223003, Jiangsu, China; b School of Business, Hangzhou City University, Hangzhou, 310015, Zhejiang, China; c School of Public Affairs, Zhejiang University, Hangzhou, 310058, Zhejiang, China; d Business & Tourism Institute, Hangzhou Vocational & Technical College, Hangzhou, 310018, Zhejiang, China

**Keywords:** Industry-education, Big data, KNN algorithm, Fuzzy algorithm, Inter-technology information management

## Abstract

The integration of big data technology in the manufacturing process has become a norm, and as society's dependence on the digital economy increases, colleges and universities must adjust their teaching methods to cater to their students' needs. In evaluating the success of business-school partnerships, there is a need for common criteria and visualising data analysis results. However, the current educational approach presents some challenges, including a lack of practical experience with software, overemphasis on theoretical concepts, and inadequate training in problem-oriented statistical modeling and big data statistics projects. Industry-education cooperation should be leveraged to enhance the implementation of big data technology and promote its overall development. This paper analyses the shortcomings of traditional talent training models in higher education and proposes incorporating industrial education to address the gaps. The paper aims to bridge the industry-education gap by developing and implementing an Inter-Technology Information Management (ITIM) system for quality education. The ITIM system uses a fuzzy algorithm to evaluate the quality of education and provides various intelligent functional modules, such as group management, financial management, and process-to-process communication. Compared to other integration models, the proposed management system offers superior performance with an industrial education performance accuracy of 98%, an average analysis, and calculation time of 20 ms and a maximum performance efficiency of 98%.By incorporating dynamic analysis of industry education, the experimental results of the talent training model have led to improvements in teaching effectiveness, student learning, and theoretical-applied teaching quality.

## 1 Introduction

Big data, fuelled by rapid advances in communications and information technology, is one of the fascinating technologies that has the potential to fundamentally revolutionise the mining business [1]. Data collected from machines, equipment and operators at every stage of production is perfectly acceptable for extensive data analysis in the manufacturing sector. Education and industry are also inseparable from big data [2]. In contrast to the important source of education, the industrial system, the educational system is responsible for the synthesis and dissemination of practical knowledge in production [3]. The identified traditional problems related to software courses and practical teaching on big data system platform need to be explained in more detail. Traditional problems include, firstly, that many software courses treat theoretical teaching as separate from student practice. Students spend a lot of time learning concepts that they may never use. There is less time for actual practice. Second, students don't receive enough practical teaching on the big data system platform, and there is no centralised practice base, which makes it difficult to guarantee that students' extracurricular activities will flourish. Third, there is a lack of training in big data statistical projects and problem-focused statistical modelling. If big data is to play a role in the decision making process for the integration of industry and education, it needs to be collected, handled, processed and organised quickly [4,5]. To improve the intelligent management platform for industry-education collaboration, we refined a KNN algorithm model for data collection. Technical support is provided for the composite optimisation of big data technology, Both education and industry functions are integrated, analysing the importance of education and industry, which shows the improved outcome results. and the study helps accelerate the development of industry-education collaboration initiatives [6].

**Fig. 1:**
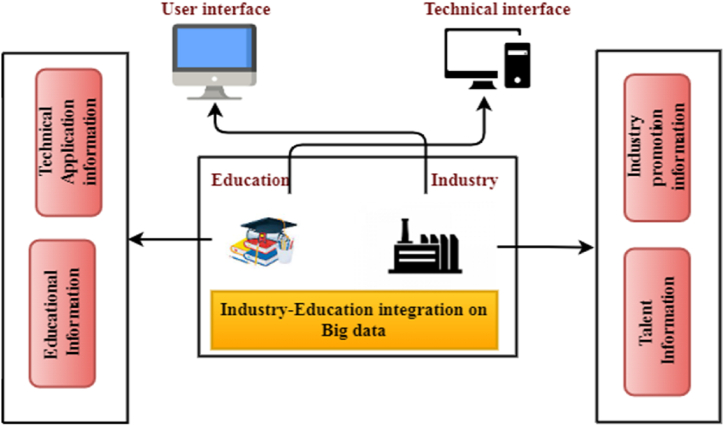
Industry-Education integration based on Big data.

Fig. 1 shows the general structure of industry education based on big data, an improved and innovative platform for managing business-academic partnerships through big data. Globally, two-way data can help promote social industry systems and improve the efficiency of education [7]. Big data technology is rapidly updated, and the data thinking and data technology brought by big data technology are reconstructing many fields and industry ecology, including education. It gives the idea of the interface between industry and education sectors to exchange talent and technical information to improve the quality of education and industry [8]. The education-enterprise collaborative education platform aims to improve talent development, promote a cooperative classroom atmosphere, and foster school-enterprise relations [9]. To this end, efforts are being made to reform talent training, build a collaborative education system, create a cooperative education pathway, promote a collaborative classroom culture, and forge links between schools, universities and enterprises to better prepare students for the workforce [10]. The dynamic mechanism of the ITIM system includes the function of group management, financial management and process-to-process communication. A university-level management information system can be created by using a three-module functional innovation management system to integrate industry and education. Management theory and practice and the manager's skills are fundamental to any thriving business in the group administrator.

The ITIM system can visualize the dynamic data of students and provide it to teachers and students in a timely manner. Student development is not static, but a dynamic process that adapts to the learner's environment and the needs of the time. It constantly pushes the person forward. It reshapes society according to the needs of the moment. Gifted people are often individuals with high levels of analytical ability [11]. These people are talented in many ways, including their ability to do their scientific study, their willingness to work with others, their ability to work in a team, and their talent for doing scientific research with other talented people [12]. Money, equipment, laboratories, libraries and other facilities and materials used in scientific research are all considered assets. Then, using the mathematical structure underlying fuzzy theory, students are asked to rate their professors on a scale from “excellent” to “good”, “fair” to “qualified” and “unqualified” [13]. Virtual reality (VR) technologies and 3D virtual representation on the web allow a more detailed presentation of educational outcomes. Excellent education is crucial to the development of creative minds in regional universities, and its promotion and practical usefulness can be supported by research [14].

The main contributions of the article are as follows.

1. The proposed method introduces the dynamic analysis of integrating industry and education by implementing an Inter-Technology Information Management (ITIM) system to improve the quality of education by implementing big data technology using the KNN algorithm.
2. The method uses functional modules such as group management, financial management and process-to-process communication, developed using a three-dimensional innovation management system to integrate the sector and education, and a fuzzy algorithm to evaluate teaching quality.
3. The results of a dynamic analysis fusion experiment between industry and education have led to improvements in training effectiveness, performance integration and data accuracy. Various training assessment datasets were used to test the proposed technology.

## 2 Literature review

AllanKlingstrom (1987) defined the integration of production and education as a talent training model, in which education and production are closely combined, engineering practice is closely combined with professional learning, and the main purpose is to serve the socie [15]. Zhang Yun and Guo Bingyu (2017) believe that the integration of industry and education in China has entered the 2.0 era, which means the deep integration and interaction of education and technology [16]. Joel Yager (2011), Cole (2011), KariLaine (2015) and other scholars believe that colleges and universities should establish corresponding industries based on their own advantages in majors, and provide experimental bases and internship positions for teachers and students based on school-run industries [[17], [18], [19]].

Wang et al. [20] introduced “one goal”, “two bases” and “three structures”, which make up the “123″ system and practice model. As the social economy and enterprise development, more creative applied talents are needed. Colleges and universities must commit to a programme of continuous and comprehensive transformation, strengthening their engagement with business and integrating business into the classroom. It is not enough to improve theoretical education; researchers must also train practitioners. Wu et al. [21] propose a merger of industry and education as a way of potential development, with consequent reforms to the talent training system in higher education. Let us assume that business universities and colleges are serious about developing the next generation of economic and social leaders. If so, they must address the urgent challenge of adapting their teaching methods to the changing demands of the digital economy and society. This study looks at the evolution of the approach to talent development through the integration of industry and academia, which has led to a re-evaluation of the traditional model of talent development in academia. The study is a breakthrough in analysing the difficulties faced by higher education institutions while taking into account new developments on the demand side for economic skills. Zhang et al. [22] analyse the core competencies of the digital media major in the light of industry needs. Investigate emerging professional characteristics in collaboration with industry and university faculty, and develop the field's distinctive advantages to inspire students to enter the field as full-fledged professionals. Enhancing students' employability and helping them to compete in the global economy. From the perspective of integrating education with industry, the curriculum system must be systematic and dynamic, adapting to the needs of the ever-evolving industrial structure. Most importantly, there needs to be close communication and cooperation between business, industry and schools, and between theoretical and applied learning. Muqian et al. [23] proposed an evaluation model of cooperative university education from a comprehensive perspective. The model uses big data to investigate the fundamental nature of global entropy in collaborative education. An investigation of the “structural function” of the regional innovation system reveals the dynamic evaluation of the collaborative university education system. The experiment was conducted to learn more about the process. Special emphasis is placed on cooperative education at colleges and universities. The research expands and extends the use of cooperative education in tertiary institutions such as colleges and universities.

Foreign universities have carried out research and development on the systematization of management information in an early period, starting from about the 1990s of the 20th century, and gradually promoted the systematization, digitization and informatization of management information, and formed a certain trend [24]. According to statistics, more than 80% of American colleges and universities have their own campus information system, which provides various functions including course service, student file management, and teacher-student communication [25]. LI Xiang-Dong has made relevant research on combining enterprise engineering posts with professional talents, designed and implemented a network training management system based on ASP.NET, which enables higher vocational colleges to conduct one-on-one talent training through relevant requirements of enterprises, and also track students' learning. Realize the information management of higher vocational colleges [26]. In the past 10 years, with the rapid development of network information technologies such as big data, mobile Internet and cloud computing, as well as the popularity and intelligence of mobile terminal devices, the campus wired and wireless integrated network has been formed to achieve standardized, efficient, intelligent work flow and in-depth mining of effective data information. Provide support for unified and centralized intelligent management and scientific decision-making in universities [27]. With the rapid development of Internet and mobile communication technology, the construction technology of college student information service platform is constantly updated and iterated. (Li Li, 2018) Big data mining and storage technologies are also widely used in colleges and universities to provide personalized information services [28].

The rapid development of Internet technology makes the function of information management system more and more rich, information management system has no longer simply refers to office automation, but represents the representative of the entire office information. All kinds of efficient and intelligent functions are added to the information management system. More and more units have adopted the office system to complete information sharing and office automation, while using the information management platform to provide users with a common platform for knowledge sharing, office convenience and information exchange.

### 2.1 System methodology

#### 2.1.1 Structure of dynamic mechanism of the industry –education integration based on big data

**Fig. 2:**
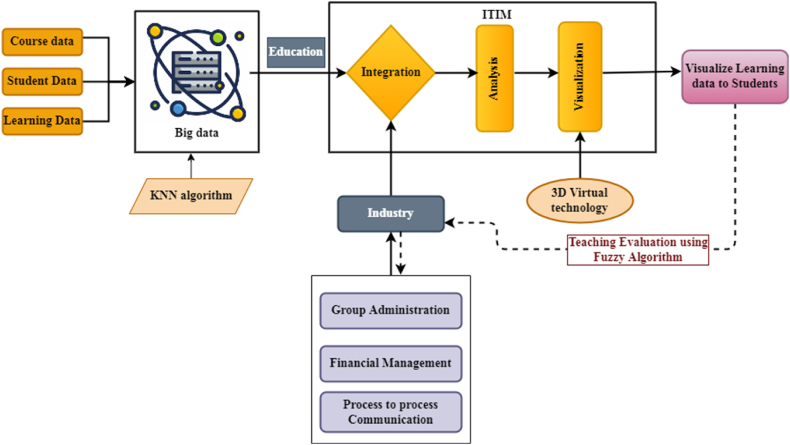
Overall Structure of industry –education integration based on Big data.

Engaging the business sector and academic institutions is a powerful technique for creating talented professionals. As the era of big data grows, so too must the corresponding educational offerings. Big data has significantly broadened the field's study subjects, and the application area has created several new technologies and business techniques that have influenced the field's development. Fig. 2 shows the overall integration structure of industry education based on big data. Practical education programmes better equip workers to meet societal demands by incorporating big data mining, analysis and processing. The main goal of practical education is to engage students and help them learn essential skills. It helps them understand and remember what they are learning. In the context of big data, how can we best educate future data analysts and other applied professionals for use in industry and for the public good? Collecting, storing, searching, sharing, analysing and visualising data are all challenging activities. The type of data can be heterogeneous, containing both organised and unstructured varieties. A new analytical paradigm has emerged. Collaboration between industry and education means that both sectors can move forward simultaneously. Collaboration between industry and education at the micro level ensures that students receive quality training by adapting their academic programmes to the needs of local businesses.

More and more people are adopting new technologies that can improve their lives by increasing practical knowledge. The level of cooperation between enterprises and academic institutions can be increased through technological improvement. Fig. 1 shows that as the informatisation of education progresses rapidly, big data technologies will provide crucial support for university education policies. As a result, the two disciplines are now more intertwined than ever before. Higher education institutions that focus on vocational training can benefit significantly from the use of big data technologies by strengthening their ties with local industries and developing new approaches to preparing the future workforce. Therefore, a network platform for vocational training built using network infrastructure is an efficient method for sharing information and promoting collaboration. The pace of technology development, including data integration, analysis and visualisation, can be accelerated by creating an intelligent management platform, ITIM, for industry-education collaboration. Despite the immaturity of the technology, it has received significant technical support from the academic community. Innovative service platforms can promote network-wide communication between primary and substations. Instructional data includes course data specifying teaching materials, student data including the total number of students, their ID, age, gender, classroom scholarship details, and learning data, all of which are obtained from big data using the KNN algorithm.

As a result, the main contribution of the study is the design of a mining-based sophisticated management system to promote inter-institutional collaboration. To facilitate the industry-education partnership of the ITIM platform, we developed a KNN algorithm model for data collection. Technical support is provided for the beneficial optimisation of big data technologies, and the research contributes to the rapid growth of industry-education collaboration methods. Business includes process-to-process communication, financial management and group management process. Both education and industry functions are integrated, analysing the importance of education and industry, which shows the improved outcome results. The learning data is given to teachers for visualisation to establish effective teaching methods, and here individual students are given learning data effectively to cultivate self-directed learners.

They integrate manufacturing and teaching, go beyond established approaches to practice, and bring students together. Bringing together academics, trainers, technicians and business leaders can break the paradigm of traditional education. By facilitating the exchange of information, educators increase the effectiveness of the relationship between education and business and develop creative approaches to education. Encourage student innovation and entrepreneurship while creating “dual-qualified” educators. The proposed method uses the fuzzy algorithm to find the best teachers for teaching. And the information technology system uses virtual reality technology for hands-on learning for students in the natural environment.

#### 2.1.2 Big data using KNN algorithm

The KNN algorithm is a common technique used in various industries, including business and STEM (science, technology, engineering and mathematics) education. The search for K-nearest neighbours is the most essential and computationally expensive part of the technique. Exploiting the power of high-performance technology is crucial, as most state-of-the-art work aims to perform the procedure in real time, or at least with reduced computational efficiency. There are several scientific applications for the massively parallel architecture of these devices, which exploits data parallelism. There are several possibilities for data pre-processing, such as data integration, cleansing and selection. As a result of the use of such data preparation tools, the synthetic quality of innovation in valuable services has been dramatically improved, the time required for collaboration has been reduced, and the feasibility of actual data collection has been increased. Unified rectification of imperfect, noisy and inconsistent real-world information is what data filtering is all about. Methods of data prediction, noise reduction and data infill improve data quality and use. Consistent data structure, verification of the absence of anomalous data, deletion of erroneous data and completion of empty data are all expected results of data cleansing. Organisations are increasingly turning to data integration, or the coordinated handling of disparate data sources within large data sets, to maximise the usefulness of information storage. Data transformation is required when the knowledge acquisition algorithm requires a different data format. Data integration involves bringing together information stored in separate databases. Selecting the right data involves filtering out the irrelevant and picking out the important.

**Fig. 3:**
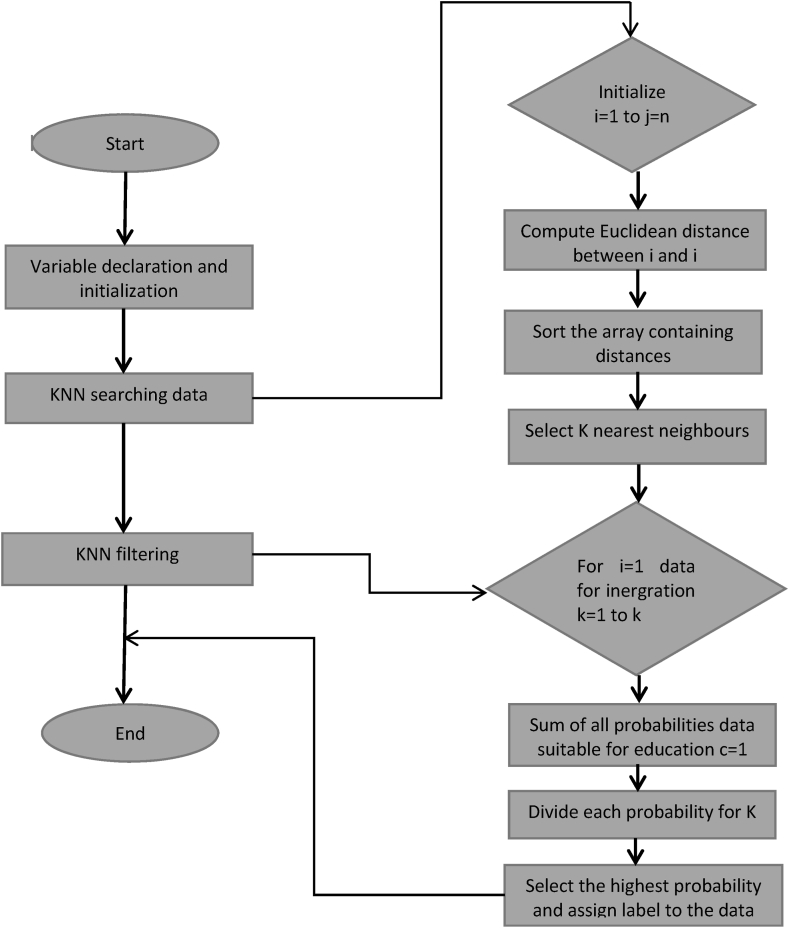
Flow chart representation of KNN algorithm used in Big data.

Fig. 3 shows a flowchart representation of KNN, information extraction and classification techniques for large datasets used in education. The KNN based algorithm approach takes as input the information generated by the information technology. The application of KNN algorithm in industrial education is novel. All matrices, hierarchies and parameters should be declared and initialised before any calculations are performed. For each dataset, two types of systems are defined: feature matrix nodes, which store the parameters used to calculate the Euclidean distance using equation (1), and feature distance, which preserves the spacing and indexes of these pixels. The Euclidean distance is calculated with i and j as the two points in Euclidean n-space, jk and ik as the Euclidean vectors.

(1) $$d\left(i,j\right)=\sqrt{\sum_{i=1}^{n}j_{k}-i_{k}}$$

The second stage is the K-nearest neighbour search. The program considers each pixel in an image, text or data set and calculates the distances between each value and each pixel in its window using the Euclidean metric. After recording all the spaces in the feature distance structure, the approach uses the merge sort algorithm to arrange them in descending order, finally selecting the K indices of the data with the shortest distances. At this stage, the window size parameters are modified based on the location of the data. Once all the information neighbours have been identified, the KNN filtering can begin. The program does this by considering the probability maps of c and marking relevant information accordingly. Before the information is processed, the probabilities of all the data surrounding the target data are summed and assigned to the appropriate class. The total sum is then divided among its K nearest neighbours. The algorithm then assigns the highest of the four optimised probabilities to the class label of the relevant data. The decision of the KNN algorithm is illustrative of a class of computational algorithms that perform general linear classification of data via supervised learning; its boundary is the most significant margin horizontal angle that solves the training inputs.

Therefore, at its core, it uses a linear classification technique with the following input data and learning objectives I=(i_1_,i_2_ … i_n_) and J=(j_1_,j_2_ … j_n_). In the feature space containing the input data, a classification model acts as a selection barrier and determines whether a learning objective is positive or negative. If any sample has a moment in time distance greater than or equal to one, then the following equations can be used to determine the maximum margin in equation (2) and the moment in time distance in equation (3).

(2) $$w^{T}I+H=0$$

(3) $$J_{i}\left(w^{T}I_{i}+H\right)\geq 1$$

where w ^T^ is the hyperplane's average vector, H represents its intercept. The range has positivity for all samples above the top border and minima for all data below the lower limit. The loss function of the algorithm is calculated as loss(P) using the probability P; 0–1 loss function cannot be used to solve optimisation problems since it is not continuous.

given by equation (4) is provided by

(4) loss(P)=max⁡(0,1−P)

The KNN algorithm is a crucial component of the big data technology stack. Our primary goal in this research was to construct and optimize the KNN algorithm for use in an inter-management platform that facilitates collaboration between the business and academic communities.

#### 2.1.3 Inter-Technology Information Management platform to integrate education-industry

**Fig. 4:**
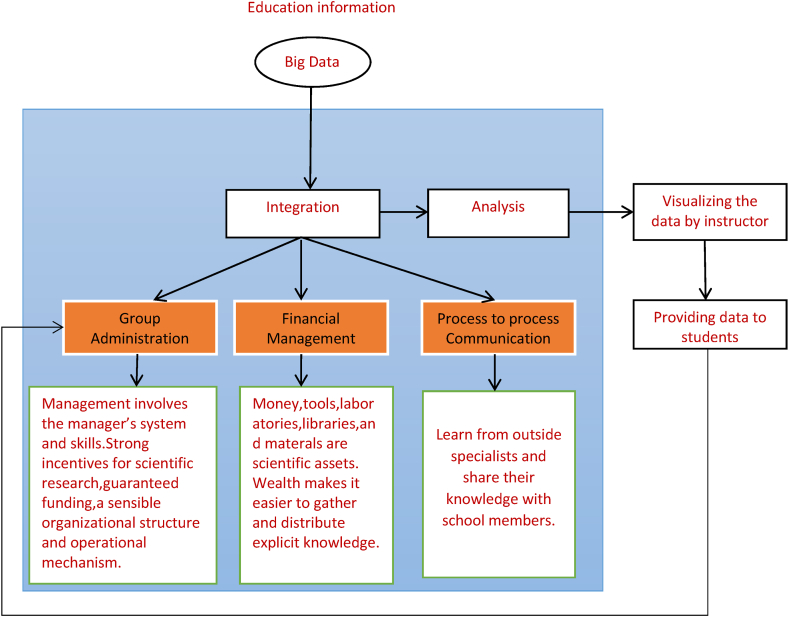
Inter-Technology Information Management Platform function modules.

Fig. 4 shows how greater collaboration between institutions and businesses can improve professional performance and public image. Utilise all educational technology resources and establish a unified management structure to ensure the continuous development of powerful technological resources. The quality of asset education in higher education can be improved by focusing on talent development, adopting market-oriented educational and institutional structures, and developing market-oriented professional infrastructure. There is a need to strengthen cooperation between academic institutions and business, with universities seeking the support of business and providing more direct support for business needs. The success of both universities and companies depends on their ability to work together to develop a “new approach to industry-university-research cooperation”, which emphasises the integration of different technologies, the involvement of teachers and students, and the practical application of research results.

Based on the ideas of “integrating production and education, taking the needs of the knowledge economy as the guide, continuous improvement as the core, and reform and creativity as the driving force”, the project aims to change the paradigm of talent training. Through restructuring, research and problem solving in effective teaching that affects the quality of talent training, promoting teaching results, getting better experiential learning and raising the overall level of operation, the university should work towards creating a new model of entrepreneurship cooperation and collaborative emergence. The proposed technology has three functional modules: Group Management, Financial Management and Process-to-Process Communication. The ITIM will have the following responsibilities for the management of education and technology: Firstly, create a brand new database for industry education by using a search engine to explore the relevant book and news information databases for cutting-edge research and development. Second, determine which areas of study will flourish in the institution's environment and how to conduct scientific studies in new ways by analysing the new technology and science database, the university's talent situation and available resources.

A university-level information system for managing the merger of education and industry is achievable on the basis of the dynamic analysis of the system of process innovation for the two processes mentioned above. The following essential components should be included in the system: A module to facilitate communication between different processes. The process-to-process management module is responsible for identifying, developing and promoting current employees, while the management module is responsible for finding, evaluating and bringing in new talent from outside. Analysis provides insight into both individual and group performance. Teachers can learn more about student interests through statistical analysis of student grades. When applied to the context of innovation, in the process of process communication, the project can act as a basic guideline, educational content can be deconstructed into modular components, and everyone benefits. The operational process of the project could serve as a source for the study question, allowing for a simultaneous theoretical and practical analysis. Students specialising in design at higher vocational schools can create a form of online teaching that takes into account the current state of the field, using economic inputs and interdisciplinary specialist research on expertise and achievements.

Module for managing one's own financial affairs: Includes two modules: one for managing the institution's internal funds and equipment for scientific research, and another for finding and using resources outside the institution. The coordinated effort between industry and academia aims to produce a workforce capable of meeting the social and economic demands of the Significant Data Age. The processes of knowledge production and talent development are organically linked through the combination of science and education.

Module for managing a group of people: The package includes integrated modules for managing scientific research and wages. Several components, such as those for the management of scientific studies, are subsumed under the overarching concept of “scientific research governance”. These include the application and approval process, the submission of science and research outputs, the evaluation of these outputs, the search for new technologies and the tracking of how well different technologies complement each other. Payroll management, programme approval and the presentation of scientific research results all fall under the umbrella of managing the day-to-day operations of an organisation. It is necessary to carry out research and analysis in other modules before drawing conclusions, such as evaluating scientific research and searching. Students can acquire and apply basic skills in all subject areas and, through an integrated approach to teaching, develop favourable learning dispositions that will serve them well throughout their education. Industry-education cooperation in universities has been a breeding ground for reform and development in the field of dynamic design education for some time. Industry-education integration in universities is beneficial to educators working to reform practical education because it provides a framework for standardising, designing, inventing and practising new approaches to teaching, while increasing opportunities for fruitful student-faculty interaction. The integration of a dual teaching group into the teaching reforms that have resulted in higher quality education and more efficient talent output has been incredibly beneficial to the field of system design.

After the integration of industry and education, the learning data helps to practically understand the importance of education and gain industry knowledge, and it's helpful for their future. The learning data is visualised on the indicator using 3D Virtual Reality (VR) technology. VR in the classroom helps students gain a fresh understanding of established topics. Students benefit greatly from being able to visualize what they are learning, especially when it comes to understanding more abstract ideas. VR is useful in education because it increases students' ability to remember what they've learned. Being fully immersed in a digital environment has been shown to improve spatial cognition and memory. That's because your brain associates the information with the physical features of your environment. Increase the depth and breadth of a system based on university-industry interaction and dynamic innovation practice education in real-world communication, focusing on raising the bar for excellence in dual innovation education through academic collaboration. Incorporate cutting-edge technology and enterprise knowledge into the professional education system, train industry talents at all levels (including curriculum design, professional co-construction, university cooperation, etc.), and conduct teacher training and applied scientific research in tandem. The high-quality training of talents and the thorough integration of the university's enterprise resources in the field of informatisation application of a specific industry have led to the university becoming the enterprise's application development partner.

#### 2.1.4 Fuzzy evaluation algorithm

**Fig. 5:**
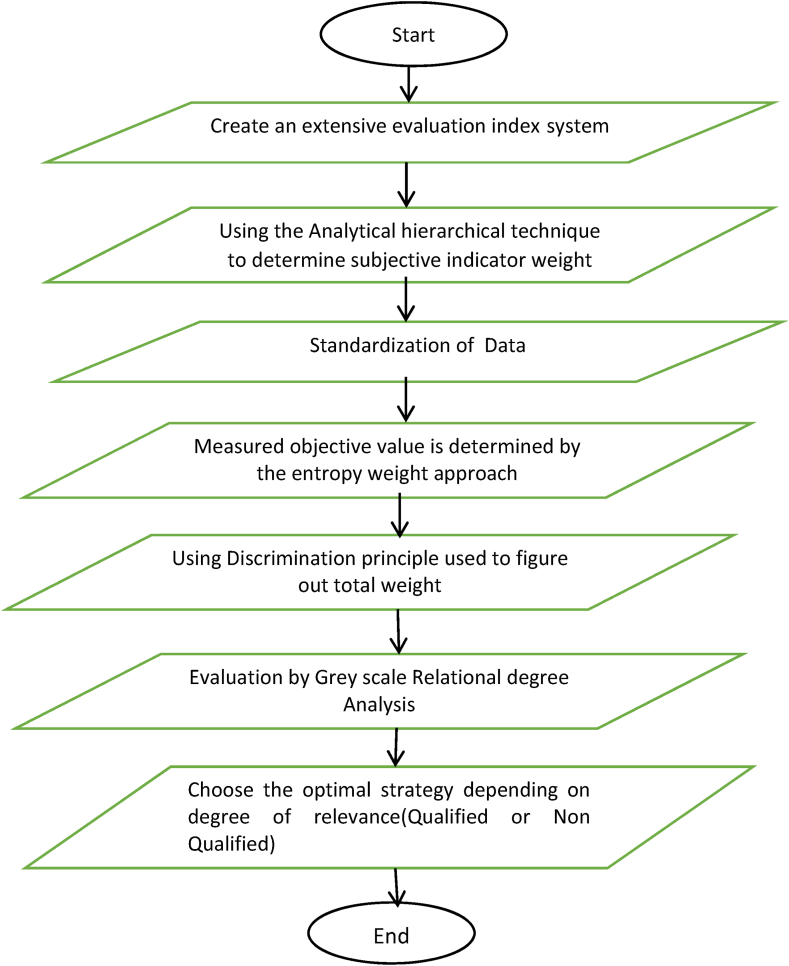
Flowchart representation of Fuzzy algorithm.

The technique is considered problematic due to the uncertainty of the classification at the transition point between the differences in the objective items. Students are asked to rate their teachers on a scale of “excellent”, “good”, “fair”, “trained” and “untalented”. Fuzzy mathematics uses rigorous mathematical approaches to describe and characterise a wide range of vague ideas and phenomena in the real world, allowing for more precise processing. Fuzzy exhaustive evaluation algorithms can now have a theoretical foundation thanks to this paper. The operation of the fuzzy comprehensive evaluation method The result of the institution's evaluation of the effectiveness of professors in the classroom is based on student feedback on a variety of factors. The result is a vague assessment of the quality of teaching at universities. The flowchart of the systematic evaluation algorithm is shown in Fig. 5 below.

Firstly, the factor (index) set evaluation (grade) set of the evaluated object is determined; Then the weights of each factor and their membership vectors are determined respectively, and the fuzzy evaluation matrix is obtained. Finally, the fuzzy evaluation matrix and the weight vector of the factors are carried out fuzzy operation and normalization, and the fuzzy comprehensive evaluation results are obtained [29].

The first step is to find the set U of evaluation factors, which is the usual exhaustive evaluation technique. Finding the typical comprehensive evaluation algorithm is the same as finding the set of comments V of evaluation criteria. Choosing a set of comments to use as scoring criteria is an alternative second step. The next step is to select the proportional weight to be given to each criterion. Solving the weight vector W= (w_1_,w_2_ … w_n_)of the evaluative component can be approached in several ways, including using the mean method, the AHP method. If the magnitude of the weighted component is greater, it has a greater significance in the overall evaluation. The weighting factor is given by equation 5

(5) $$\sum_{i=1}^{n}w_{i}=1$$

Using the Weight factor W and evaluation matrix *X* = *(x*_*ij*_*)*_m*n_ is the i-factor unidimensional analysis and have the degree of the member of j th element of standard set V. And calculate a comprehensive fuzzy value in equation (6) is evaluated by

(6) $$F=W.X=\left(w_{1,}w_{2},\ldots \ldots w_{n}\right)\left[\begin{array}{ccc} x_{11} & x_{12}\ldots \text{.} & x_{1n}\\ x_{21} & x_{22}\ldots \text{.} & x_{2n}\\ x_{n1} & x_{n2}\ldots \text{.} & x_{nn} \end{array}\right]$$

Determine an overall evaluation score of the indicator that is being evaluated. Individuals are assigned meaningful scores after the evaluation, points to various evaluation levels, and an allocation matrix *E* = *(e*_*1*_*,e*_*2*_ *… e*_*n*_). The result with a score that is meaningful to individuals is represented by equation 7

(7) $$Y=F.E=\left(F_{1,}F_{2}\ldots ..F_{n}\right).\left[\begin{array}{c} e_{1}\\ e_{2}\\ e_{n} \end{array}\right]$$

Assessing the quality of education involves making value judgements in the light of established criteria. As a comprehensive concept, the quality of teaching encompasses many facets of education. Evaluating the performance of educators requires a dissection of the subject matter of the evaluation in the light of its objectives. The evaluation is carried out by identifying the factors that best capture its distinctive and idiosyncratic characteristics. The quality of learning is developed as a three-stage evaluation index using research methods such as expert interviews, researcher observation of students and teacher discussion, etc. The evaluation index system provides an overall assessment of the quality of the education provided, taking into account the results of the individual evaluation indices according to a predetermined formula.

## 3 Results and discussion

### 3.1 Performance evaluation

The proposed cross-technology information management platform is an innovative method to integrate education and industry for high quality learning data with high accuracy. The dataset used to train the proposed method is the Higher Education Students Performance Evaluation Dataset. Students from the Faculties of Engineering and Educational Sciences make up the 2019 sample. The aim is to predict the final grades of the students. There have been 29469 page views and 4154 downloads [30].

https://www.kaggle.com/datasets/csafrit2/higher-education-students-performance-evaluation.

The performance metrics of the proposed integration process based on the big data include the average computation time, the accuracy of various algorithms, the computation time of the big data, the performance efficiency, and the execution time. The optimised effect of the big data technology on the management platform was determined through a thorough evaluation of the designed big data-based technology using the dataset. Finally, we compared and analyzed the K-nearest neighbour (KNN) models with another model's simple and valuable classification approach, which uses distance measurements between samples to determine which K neighbours to use for classification. The essence of the technique is that a model should be classified alongside most of the K-nearest samples in the feature space. There are three steps to classification: determining which neighbours are closest based on distance calculations, finding the nearest neighbour (i.e. choosing the K-value), and finally making a decision and classifying. An intuitive classification method is the KNN algorithm.

### 3.2 Performance accuracy of industry-education based on big data

**Fig. 6:**
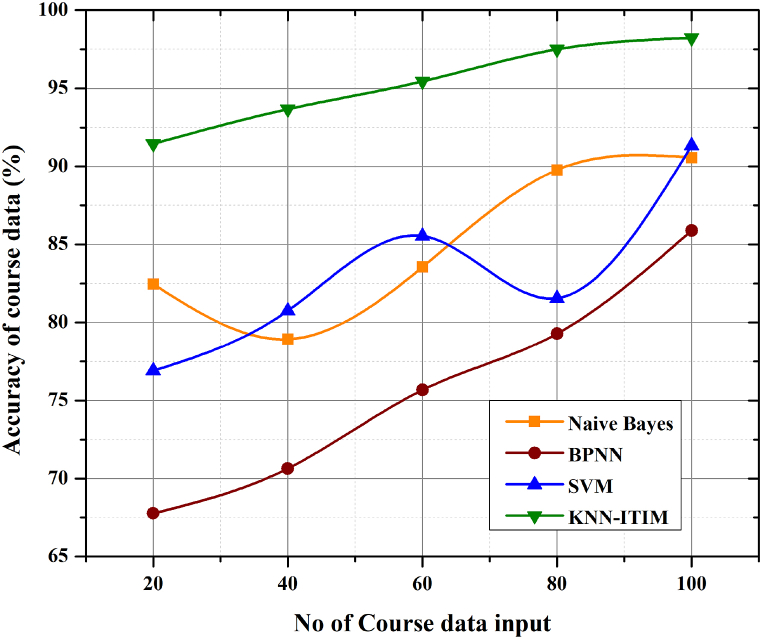
Performance Accuracy of Industry-education based on Big data.

Fig. 6 shows the graph between the number of iterations and the extraction accuracy for different types of data, which is best achieved by the best designed KNN-ITIM algorithm model. The accuracy of the KNN algorithm is calculated and compared with Naïve Bayes, SVM (Super Vector Machine) and BPNN(Back Propagation Neural Network) algorithms. Evaluation of accuracy is essential in any attempt to classify things. The next step is to compare the identified data with another reliable data set. It is possible to learn from experience, but this is both expensive and time consuming. The performance accuracy equation (8) is given by

(8) $$Accuracy=\frac{Tp+Tn}{Tp+Tn+Fp+Fn}$$

For each trial, both the total number of successes (TP) and the total number of failures (TN) are displayed. The True Negative measure represents the number of cases where the generic type was correctly identified. False positives (FPs) refer to the frequency with which otherwise innocuous signals were misidentified as threats. The percentage of attacks that are incorrectly classified as low risk is known as the False Negative (FN) rate. Since FN is generally considered to be riskier than FP, many intrusion detection systems（IDS） will make fewer FN assumptions. KNN maintained a consistently high level of accuracy regardless of the number of iterations applied to the model, ranging from a high of 98% to a low of 91%. Overall, the accuracy of KNN in the other models fluctuated and its stability was poor. The advantages of the designed model in terms of the accuracy of the KNN course data in ITIM are also evident.

### 3.3 Average computation time (ms) of big data analysis

**Fig. 7:**
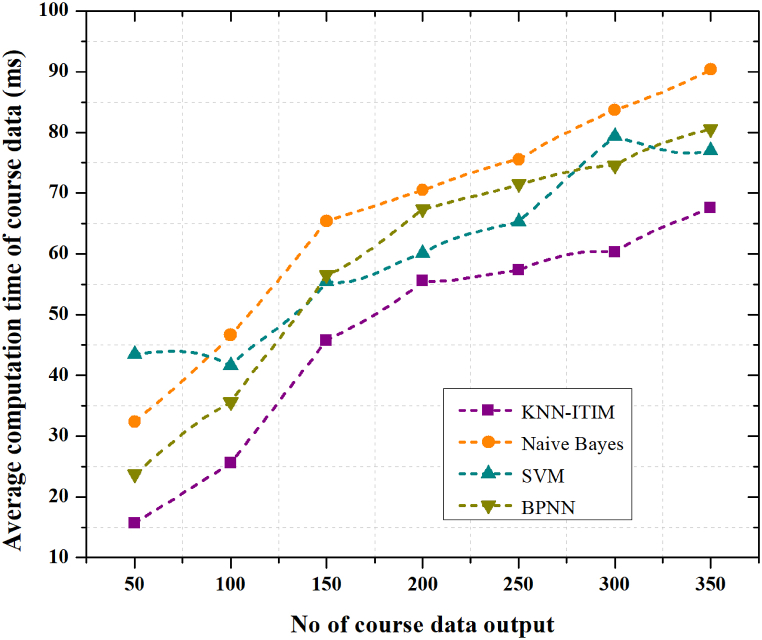
Average Computation time (ms) of Big data analysis.

Compared to the other methods, the results obtained by the ideally constructed ITIM platform using the KNN algorithm are significantly superior. Overall, after about 100 significant data outputs, including classification of student data, course data and learning data of the specified KNN algorithm model, the average computation time tended to be stable with a maximum of about 95 ms. The average computation time was about 20 ms. The results showed that the model pair in KNN is optimised using big data. The results of the evaluation of the computation time of the optimally built KNN algorithm model are compared with the other models in Fig. 7.

#### 3.3.1 Performance Efficiency(%) of ITIM technology

**Fig. 8:**
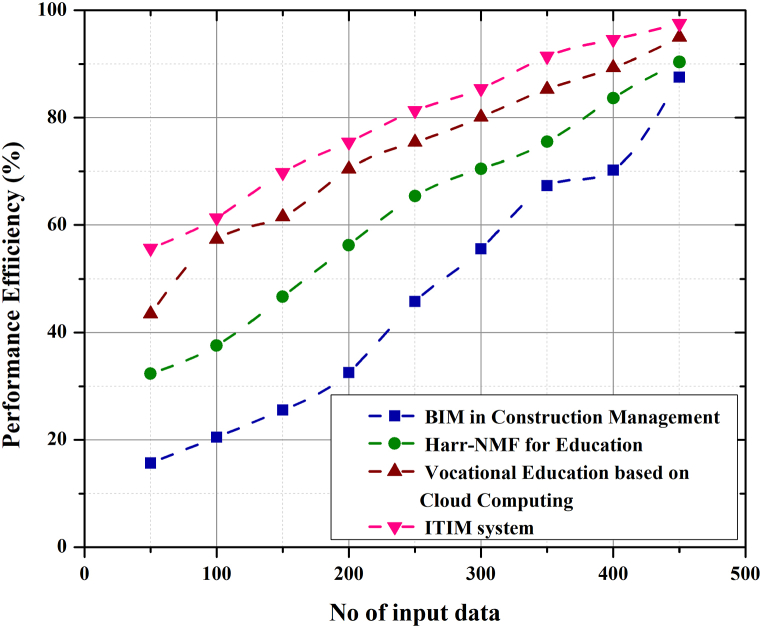
Performance efficiency of ITIM system.

The graph in Fig. 8 shows the effectiveness of using the ITIM within the proposed framework for integrating industrial education for practical training of students. Communication models make it possible to recognise and understand the links between the different components of the process. Models, symbolic representations of creative thought, and different ways of thinking about different aspects of communication can help us in the process of developing a more efficient system. The Performance Efficiency Equation (9) is represented as

(9) $$\eta =\frac{R_{o}}{R_{n}}*100$$

where *R*_*0*_ is the high-efficiency integrated output, and *R*_*n*_ is the learning data input. The proposed model is compared with the Integration BIM into construction management, Harr-NMF for education, and vocational education training based on Cloud computing technology. From the graph, the proposed management system has the highest performance efficiency of 98% compared to the other integration models. Mastering the fundamental theories, knowledge, and skills of educational technology on the idea of cutting-edge manufacturing, cutting-edge pedagogical practices, and increasing the efficacy of the proposed technology all fall under the term of knowledge and skills of educational technology.

### 3.4 Average Execution time (s) of management platform

**Fig. 9:**
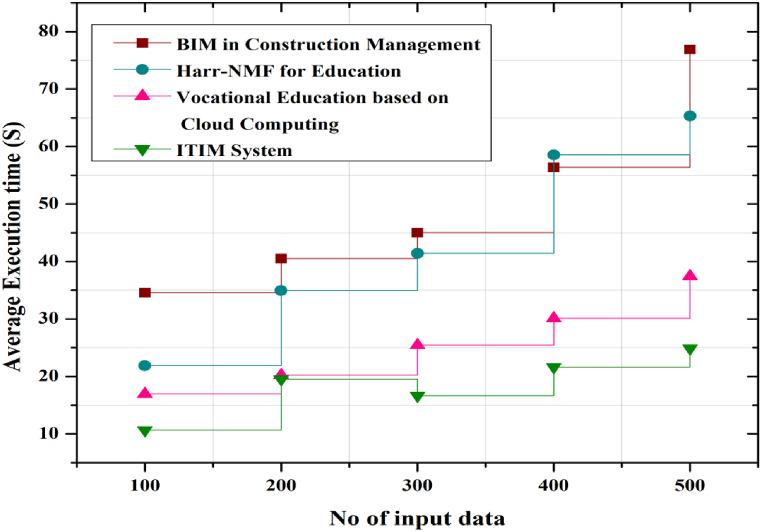
Average Execution time of ITIM system.

Fig. 9 shows the graph plotted between the execution time of the management system for the integration of educational and industrial data and the execution time. Then the information is given to the instructor visualising the learning data. For better system effectiveness in the paper, the ITIM and the running time of the system on the instructor database are plotted. Fig. 9 shows the corresponding line graphs. The time is plotted vertically in seconds, while the number of inputs is plotted horizontally as the interval between successive values of the objective function. The algorithm proposed in the study is efficient and in some respects superior, making it suitable for use in systems that integrate business and higher education. The execution time is calculated based on the output time to the input data from the big data. The proposed approach is compared with the traditional management and education models, such as the integration of BIM into construction management, Harr-NMF for education, and vocational training based on cloud computing technology. The graph shows that the ITIM method has less execution time, so the effectiveness of the system has been increased.

### 3.5 Evaluating teacher quality using weight index

The assessment on the relevance of education Quality Assessment Indexes in Colleges and Universities was distributed to expert educators and education managers. Ten responses were selected for further analysis. Once surveys were collected, they were compared to one another to determine how consistently the index importance was being measured. The weight given to each criterion for assessment is then determined using the Analytical Hierarchical Process (AHP) method and equation (10), and *x*_*ij*_ gives it as the normalized index value parameters divided by the number of criteria (n) used to calculate the weighted index. The evaluation index is based on the content teaching, teaching attitude, skills, effect, and teaching method. Table 1 below shows the survey results by how much each assessment index was given, and the weight index is calculated using equation (10).

(10) $$W_{ij}=\frac{\sum_{j=1}^{n}x_{ij}}{n}$$

**Table 1:** Evaluation of teaching based on fuzzy algorithm.

Target Layer	Standardization Criteria	Index Layer (*x*_*ij*_)	Weight Index (*w*_*ij*_)
Teaching Quality of Instructor	Content teaching(x_1_)	X_1_(0.33)	1.34
		X_2_(0.65)	
	Teaching attitude (x_2_)	X_2_(0.65)	2.45
		X_3_(1.67)	
	Teaching skills (x_3_)	X_3_(1.67)	1.37
		X_4_(0.56)	
	Teaching effect (x_4_)	X_4_(0.66)	0.45
		X_5_(0.33)	
	Teaching method (x_5_)	X_5_(0.33)	0.32
		**X**_**1**_**(0.33)**	

The assessment status of each index has been determined and is shown in the table. This data is then combined with other data to produce an overall assessment of the effectiveness of the assessment system as a measure of the quality of teaching. Based on the weight index, the teaching review is determined to be qualified or non-qualified. If the index value is less than 1, the indicator teaching is not satisfied using the fuzzy algorithm. The results of the simulation experiment further strengthen the predictive validity of the screening test, showing that students' intuitive and hunches-based assessments of the importance of elements in industrial education do not perfectly match the exam results.

## 4 Conclusion

Digital transformation progression offers substantial opportunities to accelerate the technical transition to the new era of the industrial internet of things (IIoT) [31]. This study proposed a big data-based integration strategy that combines the KNN and fuzzy algorithms to evaluate the reliability of indicators in big educational data. The proposed model aims to improve the quality and effectiveness of education by evaluating and implementing a distinctive potential training model for the convergence of business and learning. The study evaluated the performance of the proposed model using performance indicators such as average computation time, algorithmic accuracy, occupancy rate, and performance management. The results showed promising outcomes, suggesting that the proposed model has the potential to improve the quality and effectiveness of education. Meanwhile, the study also highlighted the need for further research on the practical applications of the model to increase its overall impact. As this study's limitation and future direction, future works can introduce specific key performance algorithms of digitalization as an enabler to achieve the Sustainable Development Goals (SDGs) and assess the impact of digital transformation on sustainability performance in a broader context (i.e., AI-driven alternative digitalization ratings) [32].

## Ethical approval and informed consent statements

The studies involving human participants were reviewed and approved by the Jiangsu Vocational College of Finance and Economics Research Ethics Review Committee. The participants provided their written informed consent to participate in this study. This article does not contain any studies with human participants performed by any of the authors.

## Author contribution statement

Zhenhua He, PHD: Conceived and designed the experiments; Analyzed and interpreted the data; Wrote the paper.

Lifeng Chen, PhD: Analyzed and interpreted the data; Contributed reagents, materials, analysis tools or data.

Lianqin Zhu: Performed the experiments; Analyzed and interpreted the data; Contributed reagents, materials, analysis tools or data.

## Funding statement

This work was supported by the 14th Five Year Plan for Educational Science of Shandong Province (2021ZC109); 2022 Jiangsu Province Education Science planning project (B20220292); 10.13039/501100002858China Postdoctoral Science Foundation (Grant No. 2023M733037); the China State Owned Assets and Enterprises Research Institute (Grant No. 2023GZ011).

## Data availability statement

Data included in article/supp. material/referenced in article.

## Declaration of competing interest

The authors declare that the research was conducted in the absence of any commercial or financial relationships that could be construed as a potential conflict of interest.
